# Assessment of an IMU-Based Experimental Set-Up for Upper Limb Motion in Obese Subjects

**DOI:** 10.3390/s23229264

**Published:** 2023-11-18

**Authors:** Serena Cerfoglio, Nicola Francesco Lopomo, Paolo Capodaglio, Emilia Scalona, Riccardo Monfrini, Federica Verme, Manuela Galli, Veronica Cimolin

**Affiliations:** 1 Department of Electronics, Information and Bioengineering, Politecnico di Milano, Piazza Leonardo da Vinci 32, 20133 Milano, Italy; serena.cerfoglio@polimi.it (S.C.); manuela.galli@polimi.it (M.G.); veronica.cimolin@polimi.it (V.C.); 2 Orthopaedic Rehabilitation Unit and Research Laboratory in Biomechanics, Rehabilitation and Ergonomics, San Giuseppe Hospital, IRCCS Istituto Auxologico Italiano, 28824 Piancavallo, Italy; f.verme@auxologico.it; 3 Dipartimento di Ingegneria dell’Informazione, Università degli Studi di Brescia, 25123 Brescia, Italy; nicola.lopomo@unibs.it (N.F.L.); r.monfrini001@studenti.unibs.it (R.M.); 4 Department of Surgical Sciences, Physical Medicine and Rehabilitation, University of Turin, 10126 Turin, Italy; 5 Dipartimento di Specialità Medico-Chirurgiche, Scienze Radiologiche e Sanità Pubblica, Università degli Studi di Brescia, 25123 Brescia, Italy; emilia.scalona@unibs.it

**Keywords:** obesity, functional assessment, upper limbs, IMU

## Abstract

In recent years, wearable systems based on inertial sensors opened new perspectives for functional motor assessment with respect to the gold standard motion capture systems. The aim of this study was to validate an experimental set-up based on 17 body-worn inertial sensors (Awinda, Xsens, The Netherlands), addressing specific body segments with respect to the state-of-the art system (VICON, Oxford Metrics Ltd., Oxford, UK) to assess upper limb kinematics in obese, with respect to healthy subjects. Twenty-three obese and thirty healthy weight individuals were simultaneously acquainted with the two systems across a set of three tasks for upper limbs (i.e., frontal arm rise, lateral arm rise, and reaching). Root Mean Square error (RMSE) was computed to quantify the differences between the measurements provided by the systems in terms of range of motion (ROM), whilst their agreement was assessed via Pearson’s correlation coefficient (PCC) and Bland–Altman (BA) plots. In addition, the signal waveforms were compared via one-dimensional statistical parametrical mapping (SPM) based on a paired t-test and a two-way ANOVA was applied on ROMs. The overall results partially confirmed the correlation and the agreement between the two systems, reporting only a moderate correlation for shoulder principal rotation angle in each task (r~0.40) and for elbow/flexion extension in obese subjects (r = 0.66), whilst no correlation was found for most non-principal rotation angles (r < 0.40). Across the performed tasks, an average RMSE of 34° and 26° was reported in obese and healthy controls, respectively. At the current state, the presence of bias limits the applicability of the inertial-based system in clinics; further research is intended in this context.

## 1. Introduction

Obesity is a chronic disease associated with several comorbidities such as non-alcoholic fatty liver, cardiovascular diseases, diabetes mellitus, chronic kidney disease, mental disorders, and musculoskeletal diseases [1,2,3].

From a biomechanical point of view, excessive accumulation of fat tissue may lead to increased axial loading on the spine and compressive forces on joints, causing alterations in body geometry and aberrant motion patterns, reduced joint range of motion (ROM), as well as to reduced postural control and stability, increasing the risk of falls and injuries [4], and the development of musculoskeletal diseases such as osteoarthritis [5]. Obesity thus hinders motor control and skills, negatively impacting daily life activities, such as walking [6], lumbopelvic movements [7,8], sit-to-stand transition [9,10], and upper limb movements [11,12].

Weight loss through controlled food intake and physical exercise may improve the quality of life of obese individuals [13]. However, they may be reluctant to exercise due to chronic joint pain and early fatigue [4]. For these reasons, obese individuals often struggle to change their lifestyle and they have the tendency to avoid physical activity, thus aggravating their condition [14]. Due to the wide range of functional limitations caused by obesity, it is important to safely and reliably quantify their impact to prevent further issues.

Motor assessment in obese has been primarily focused on gait analysis performed with marker-based optoelectronic motion capture (MoCap) systems within a controlled laboratory set-up [15]. Differences in terms of spatio-temporal parameters between obese and normal-weight individuals have been found, even though results are often inconsistent among different studies, mainly due to the use of dissimilar experimental protocols and the subjects’ characteristics. Regarding lower limb kinematics, the effect of body fat accumulation has been investigated also in functional tasks such as sit-to-stand [10] and stair climbing and descending [16], to detect possible adaptation strategies to avoid joint pain [17,18,19]. With respect to trunk motion, the increased axial load on the spine causes an increase in abdominal girth and a ventral shift of the center of gravity, resulting in a loss of the neutral position. As a result, thorax is ventral to the pelvis, thus increasing the forces experienced by the spine causing aberrant trunk kinematics during flexion, bending, and rotation [20,21], and static load-handling tasks [7]. Concerning upper limbs, different motion strategies during reaching tasks have been observed in obese patients [22] with respect to normal-weight subjects, and reduced upper limb range of motion has been measured in obese female workers during representative motion of common occupational tasks [23].

In the last decade, wearable sensors based on inertial measurement units (IMUs) have also been applied in motion analysis research on obese subjects, both in inpatient assessment and in daily living activity monitoring. IMUs are stand-alone devices integrating micro-electromechanical systems (MEMS) based on different tri-axial sensors (i.e., accelerometer, gyroscope, and magnetometer) to provide orientation data with respect to a local reference system. Therefore, IMUs can address different body segments to generally enable—through a proper sensor-to-segment calibration—a direct determination of body kinematics [24,25,26]. IMU-based systems are inexpensive, portable, lightweight, easy to wear and set up, and they do not require either the use of cameras or complex laboratory settings, enabling motor assessment in unconstrained environments and in daily-life contexts [27,28,29,30].

IMU-based gait analysis in obese individuals has been performed in several studies, although with different set-ups and protocols for sensors’ placement. For instance, Cimolin et al. [31] used the information retrieved from a single IMU placed on the lower back to compare the performance of specifically designed shoes for obese subjects with everyday sneakers in terms of spatiotemporal parameters during the 6 min walking test (6MWT) and a 30 min outdoor gait test. The use of complex systems based on multiple sensors has also been explored. For instance, a seven-IMU set-up has been validated against multiple systems consisting of six-foot switches and electro-goniometers [32] and then applied to compare gait characteristics in terms of spatiotemporal parameters and lower limb joint kinematics of obese and normal weight subjects during a 14 m straight walk [33]. Seven IMUs have been used also by Meng et al. [34] to measure gait features and investigate the possible relationship between body fat and gait features in normal weight, overweight, and obese subjects. A 200 m straight walking was performed, and sensors were placed on the sacrum and, bilaterally, on the front of the thighs, the shanks, and the dorsal surface of the feet. On the other hand, there is little research concerning upper-limb movements in obese individuals [15]. Nevertheless, promising results have been reported by various studies on healthy individuals in estimating upper body joint angles using IMUs.

Shoulder motion is the result of the contribution of five different joints, including scapulothoracic, humerothoracic, and glenohumeral joints. With respect to scapulothoracic and humerothoracic kinematics, Cutti et al. [35] compared IMU data with optoelectronic motion data while evaluating scapulothoracic during different single-joint-angle movements. For both joints, an RMSE of between 0.2° and 3.2° was obtained. The same procedure for calculating scapulothoracic joint angles was adopted by Parel et al. [36] when tracking scapular motion during shoulder flexion and abduction. Although higher than those reported by Cutti et al. [35], the RMSE values are in line with those achieved by Friesen et al. [37] during the execution of eight different dynamic tests, confirming the consistency of the movement patterns between IMUs and MoCap. With respect to glenohumeral joint, the highest accuracy was reported by Robert-Lachaine et al. [38] when comparing the data recorded by a full-body IMU system and by a MoCap during short movements and long handling tasks, with an RMSE below 3° and 5°, respectively.

Concerning elbow motion, various studies assessed the agreement between IMU and MoCap measurements during elbow flexion/extension and pronation/supination [39]. For both movements, an RMSE lower than 5° was reported, not only during the execution of short movements [35], but also during more complex daily-life tasks as pick-and-place and drinking [40].

Although various systems using body-worn sensors to measure body kinematics showed a good concurrent validity with respect to the gold standard, and their usage has become widespread, their accuracy and reliability need to be further evaluated. In fact, several validation studies have been conducted regarding IMUs in different clinical settings [41], but error quantification differs among studies due to different sensor designs [42], and lack of standardized protocols [43].

Despite the advantages offered by IMU-based set-ups, their use is thus not free from concern. First, it should be noticed that non-invasive and skin-mounted technological solutions may be vulnerable to soft tissue artifacts (STA) [44], particularly when considering obese individuals. Since IMUs are usually fixed with elastic bands, the motion of the underlying soft tissues between the sensor and the bone may cause a relative bone-sensor motion leading to inaccuracies in the estimation of rigid body poses and kinematics [45]. In addition, kinematic crosstalk may happen and introduce an error in the definition of the local reference frame of the body segment, thus affecting IMU measurement. These drawbacks are particularly crucial when obese subjects are considered due to their abnormal amount of fat tissue.

Despite the evidence of upper limb functional limitations in obesity, to the best of our knowledge, just a few previous studies addressed upper body motion in such conditions using IMUs. Indeed, we hypothesized that—in this specific context—the use of wearable IMUs allows for the non-invasive analysis of upper limb function in obese subjects, also considering a possible application in unsupervised environments. Therefore, the aim of this study was to validate an experimental set-up based on IMUs for upper body motion analysis in obese patients compared with respect to healthy subjects; furthermore, we aimed to evaluate the upper limb functional limitations in this kind of obese patient.

To date, various studies investigated the application of inertial-based systems to movement analysis in obese population, trying to address the presented drawbacks.

## 2. Materials and Methods

### 2.1. Participants

Two cohorts were enrolled on a voluntary basis. The first group included twenty-three obese individuals (Obese Group, OG) recruited among the patients following a 4-week multidisciplinary bodyweight reduction rehabilitation program at San Giuseppe Hospital (IRCCS Istituto Auxologico Italiano, Piancavallo, Italy), whilst the second group included thirty healthy weight individuals (Healthy Weight Group, HG). Inclusion criteria were the following: age ≥ 18 years; BMI: 20–25 kg/m^2^ for HG and >30 kg/m^2^ for OG; and the absence of neurological or musculoskeletal conditions affecting motor function and patterns. The study was approved by the Ethical Committee and carried out in accordance with the ethical standards of the Institutions and to 1964 Helsinki Declaration and its latest amendments. Written informed consent was obtained from all participants. The study took place from November 2021 to December 2022 and it involved simultaneous data collection with an optoelectronic marker-based motion capture system and an IMU-based system across a set of tasks for upper limbs.

### 2.2. Experimental Set-Up and Study Design

Optical data collection was performed with two MoCap systems. The OG was acquired at San Giuseppe Hospital using a 6-camera system (VICON, Oxford Metrics Ltd., Oxford, UK; sample frequency: 100 Hz), whilst the HG was acquired at Politecnico di Milano via an 8-camera system (SMART-DX400, BTS Bioengineering SPA, Milan, Italy; sample frequency: 100 Hz). Participants’ anthropometrics (i.e., height, weight, distance between acromion and shoulder joint center, elbow width, wrist width, distance between anterior iliac spines, pelvis thickness, leg length, knee width, and ankle width) were measured. In both the set-ups; we used the very same marker-set, which included the use of 65 spherical reflective markers (Ø = 10–15 mm) that were attached over the anatomical landmarks of each subject according to a modified version of the plug-in gait full-body [46,47], and specific 3D-printed clusters. 

Anatomical landmarks were manually identified by palpation as regions of reduced tissue thickness between bone and skin. Thirty markers were placed on the junction between the clavicles, sternum, 7th cervical vertebrae, 10th thoracic vertebrae, and bilaterally on the acromion, lateral and medial humeral condyle, laterally and medially on the wrist, lateral and medial femoral epicondyle, lateral malleolus, calcaneus and foot (corresponding to the 1st and 5th metatarsal heads). Eight 3D-printed 4-marker clusters were attached with elastic bands to the subject’s arm, forearm, thigh, and shank on both sides, whilst a 3-marker cluster was placed on the sacrum.

The proposed adjustments with respect to the standard plug-in gait models were made to attempt to reduce the STA and their related errors in the estimation of the biomechanical quantities [45]. The clusters were introduced to define a technical reference system for each segment to reduce STA-affecting markers individually placed over the body surface and to reconstruct the model in case some markers were lost during the motion capture process. In addition, the markers on the anterior superior iliac crests were not physically placed on participants’ body to avoid their shift from the ideal position, that could have occurred specially in obese individuals due to the high amount of abdominal fat. Instead, such landmarks were manually identified by an expert operator and pointed out in an additional static acquisition that was used to reconstruct their position afterwards.

Inertial measurements were collected with a 17-IMU system (Awinda, Xsens Technologies, Enschede, The Netherlands). Each IMU integrates a 3D accelerometer, a 3D gyroscope, and a 3D magnetometer and it addresses a specific body segment according to its ID and manufacturer’s guidelines. To reduce artifacts due to the use of clothing, double-sided tape was used to attach the IMUs on sternum, scapulae, feet, hands, and forehead, whilst IMUs on sacrum, upper and lower limbs were allocated in a specifically designed slot in each 3D-printed cluster fixed with an elastic band. Each participant was asked to move the arm and the tightness of the band was regulated to limit relative motion between the structure and the skin as much as possible. 

Specific anthropometrics (i.e., height, shoulder width, elbow span, wrist span, arm span, distance between anterior iliac spines, hip height, knee height, ankle height and foot length) were required by the motion tracking system to perform a two-phase sensor-to-segment calibration. Through the dedicated software (MVN Analyze software, v. 2023.0), a 3D virtual space was created to visualize the movement of an avatar moving according to the subject equipped with the IMUs. The first calibration step required the subject to stand still in an N-pose to define the relationship between each IMU and segment orientation, whilst the second step required a dynamic acquisition, during which the subject was asked to walk back and forth for 5-to-10 m. Although the experimental protocols were implemented for full-body analysis, in this specific study, for both the optoelectronic systems and the IMU-based one, we focused the evaluation only on upper limbs. 

**Figure 1:**
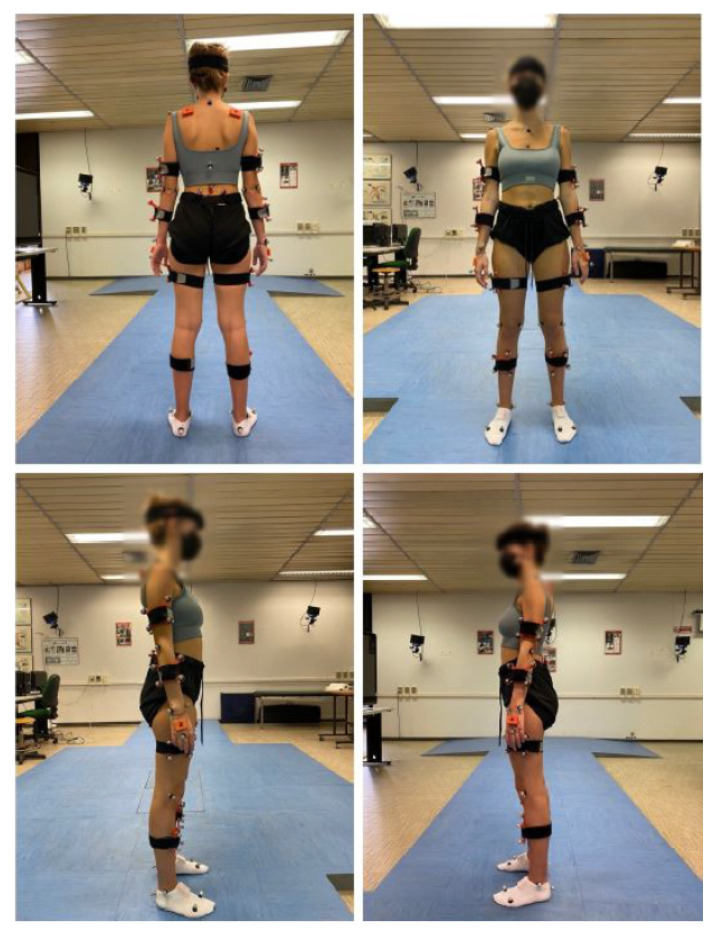
A subject (HG) equipped with the marker set and the inertial system.

A set of three tasks targeting upper limb motions were performed by each participant, simultaneously equipped with the IMUs and the marker set (Figure 1). Starting from a seated position and with arms along the sides (i.e., N-pose), the participants were asked to perform the following gestures:

- Frontal rise of the arm: Six maximal frontal rises of the arm.
- Lateral rise: Six maximal lateral rises of the arm.
- Reaching: A target (i.e., a reflective marker) was mounted on the top of a tripod that was placed at the maximum height that the subject could reach at a horizonal distance corresponding to 80% of the subject’s arm length. Starting from the N-pose, each subject was asked to reach the target for six times in their most natural way. The Range of Motion (ROM) of both the shoulder and the elbow was approximately the same throughout the task [48], and no forearm rotation was either required or noticed.

Each task was performed twice at the subject’s self-selected speed. Frontal and lateral rises were performed bilaterally, whilst the reaching task was performed only with the dominant upper limb. For the obese subjects, more prone to fatigue, we tried to guarantee their overall comfort and safety throughout the tests.

### 2.3. Data Analysis and Processing

Raw optical data were processed with SmartTracker (BTS Bioengineering, Milan, Italy) and Nexus (VICON, Oxford Metrics Ltd., Oxford, UK), respectively, and then by custom routines implemented in Matlab (version R2022b, The MathWorks Inc., Natick, MA, USA). Each 3D marker coordinate of the static acquisition was linearly interpolated and filtered with a zero-phase fourth-order low-pass Butterworth filter with a 1 Hz cut-off frequency. Local reference system, relative roto-translation matrix, and joint centers were defined for thorax, shoulders, elbow, wrists, and hands. The same interpolation and filtering methods were applied to 3D coordinates of the marker of the dynamic trials. Starting from the 3D coordinates in the global reference frame, the 3D coordinates in the local reference frame, the joint centers, and the rotation matrices were defined for each joint and transformed into their quaternion representation. Joint angles were then computed as the product between the conjugate and the proximal and the distal joint quaternion and expressed in terms of flexion/extension, adduction/abduction and internal/external rotation via an algorithm based on Euler angles. The ZXY axis rotation sequence was adopted for elbow angles in all tasks. Conversely, shoulder angles were computed using the YXY rotation sequence in frontal rise and reaching tasks, whilst the XZY axis sequence was adopted in the lateral rise. Finally, the so-computed angles were filtered with a zero-phase fourth-order low-pass Butterworth filter with a 1 Hz cut off frequency.

All the data acquired via the IMU-based system were exported via MVN Analyze Software and processed in Matlab. The anatomical frame of each sensor was retrieved from the recordings and joint angles were computed using the same axis rotation sequences and filtering approach as the optoelectronic data.

For each task, the performed repetitions were identified by detecting the position of the maximum and minimum values of the angle in each curve, focusing on the principal rotation angle involved in each movement (e.g., flexion angle in flexion movement); the first and the last repetitions were removed to avoid irrelevant variability. The ROM was computed by averaging the difference in degrees (°) between the maximum and the minimum reached during each repetition. All signals were detrended and resampled in time on a 0–100% range.

### 2.4. Statistical Analysis

ROM data were checked for normality via the Lilliefors test. Since a normal data distribution was confirmed for both groups, the variables were reported in terms of mean and standard deviation. 

The comparison between the ROMs retrieved from MoCap and inertial system was performed in terms of accuracy and Root Mean Square Error (RMSE), which were computed according to Equation (1) and Equation (2), respectively:

(1) $$Accuracy=\frac{{ROM}_{IMU}-{ROM}_{MoCap}}{{ROM}_{MoCap}}*100$$

(2) $$RMSE=\sqrt[{}]{\sum_{i=1}^{n}\frac{{\left(\hat{y_{i}}-y_{i}\right)}^{2}}{n}}=\sqrt[{}]{\sum_{i=1}^{n}\frac{e_{i}^{2}}{n}}$$

In Equation (2), *ŷ_i_*, …, *ŷ_n_* are the ROMs computed from the MoCap, *y*_1_, …, *y_n_* are the ROMs computed from the IMU (thus, *e_i_*,…, *e_n_* are the errors), and n is the number of observations (i.e., number of participants).

On the ROMs, Pearson’s correlation coefficient (r) was computed to describe the agreement between the two systems. In addition, the level of agreement (LoA) between the measurements was graphically assessed via Bland–Altman (BA) plots. 

Finally, a two-way ANOVA test was applied on ROMs considering “patient” (OG or HG) and “system” (MoCap or IMU) as factors. Whenever significant, multiple comparisons with Bonferroni correction were performed to highlight if the presence of a possible functional limitation in the motor pattern was detected by both systems.

In addition, the waveforms of the signals were compared via a one-dimensional statistical parametric mapping (SPM) based on a paired *t*-test.

## 3. Results

The demographic characteristics of the involved populations are reported in Table 1.

A preliminary unpaired t-test was applied to compare right and left ROMs in frontal and lateral rise. Since no significant difference (*p* > 0.05) was found, the two sides were pooled together. Table 2 reports the mean and the standard deviation values, together with the results of the comparison in terms of ROM and the agreement analysis between MoCap and IMU-based systems. Since the tasks mainly involved shoulder motion, it was decided to focus on shoulder joint angles. In the reaching tasks, the elbow’s motion was also considered. 

Regarding HG, the ROM values appear to be coherent between the two systems for shoulder and elbow flexion/extension, whilst a difference of about 20° can be observed for shoulder abduction/adduction. Strong correlations were found for shoulder flexion/extension in the frontal lift, for elbow flexion/extension during the reaching, and for shoulder abduction/adduction in the lateral lift. Concerning the obese group, the ROM values show a mean difference of about 19° between the two systems. Pearson’s correlation coefficient values (r) range between 0.40 and 0.66, so only moderate correlations were found.

The results of the two-way ANOVA are reported in Table 3. In all tasks, statistically significant differences were found between the shoulder ROM of the two groups, whilst no differences were found in elbow flexion/extension. Multiple comparisons with Bonferroni correction (Table 4) report that the inertial system was not able to detect a significant difference between groups in shoulder abduction/adduction in the lateral lift as the MoCap. For the other variables, the results indicate agreement between the two systems.

**Figure 2:**
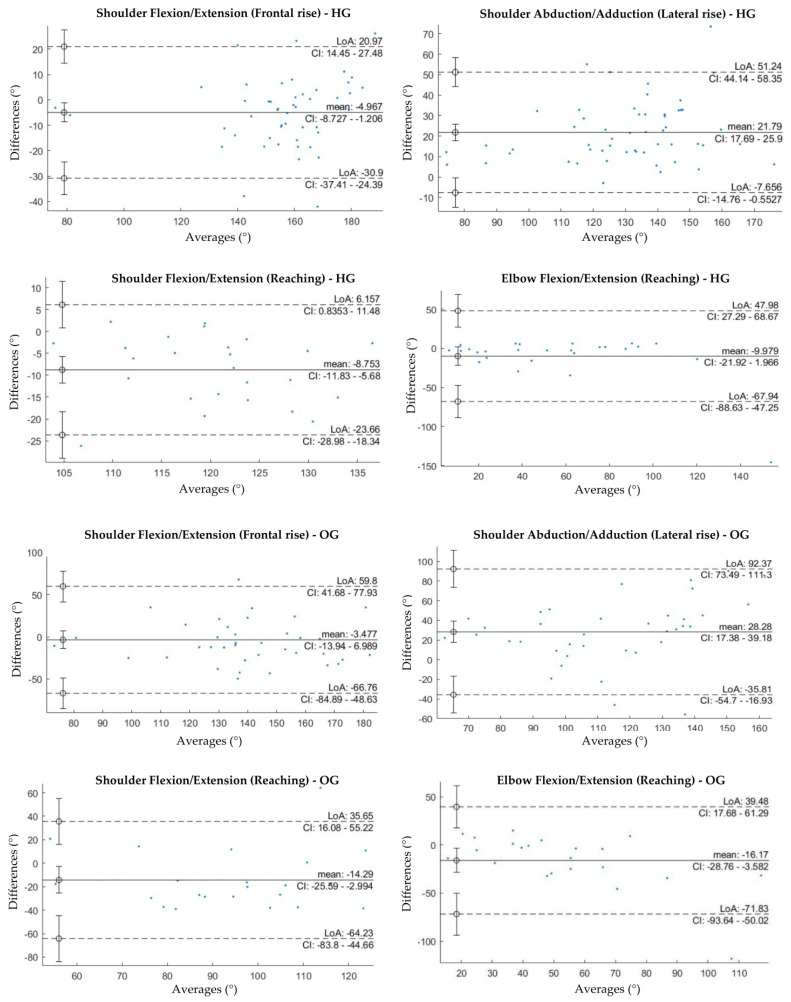
Bland-Altman plots of the average value of ROM retrieved from the MoCap and the inertial system plotted against the difference between the two systems for each group and motor task. Only BA plots for the principal rotations of each task are reported.

Bland–Altman plots for all the estimated variables are reported in Figure 2. Bland–Altman analysis is a graphical method to evaluate the agreement between two paired values and to verify where the 95% difference falls. In each plot, the horizontal lines represent the mean difference and the LoA, defined as the mean difference ± 1.96× standard deviations. The differences between the two paired values are reported as y-values whilst their averages are displayed as x-values. In the current analysis, a globally good association between the measurements of the two systems for both groups could be observed, despite the presence of some bias. In fact, BA plots for principal angles of each task reported the presence of a bias for both groups. The lowest biases were obtained for shoulder flexion-extension in frontal rise (HG: −4.967°, OG: −3.477°), whilst the highest values were reported for shoulder abduction/adduction in lateral rise (HG: 21.79°, OG: 28.28°). All BA plots had wide limits of agreement, especially for the obese group.

**Figure 3:**
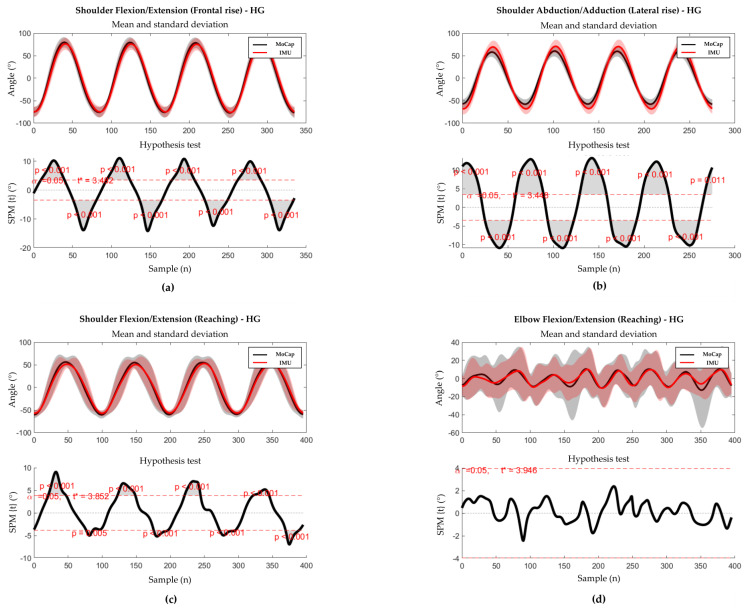
Results of the SPM analysis and the hypothesis test for HG. Thick lines represent the mean curves, with shaded areas representing the standard deviation. The analysis was performed only on the principal angles of each movement. (**a**) SPM analysis (up) and hypothesis test (down) for shoulder flexion/extension during the frontal rise. (**b**) SPM analysis (up) and hypothesis test (down) for shoulder abduction/adduction during the lateral rise. (**c**) SPM analysis (up) and hypothesis test (down) during the reaching task. (**d**) SPM analysis (up) and hypothesis test (down) for elbow flexion/extension during the reaching task.

**Figure 4:**
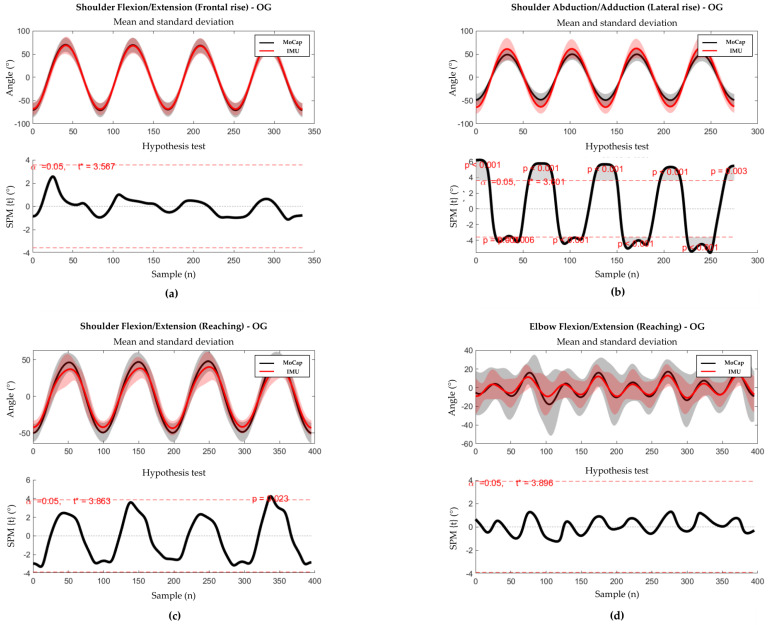
Results of the SPM analysis and the hypothesis test for OG. Thick lines represent the mean curves, with shaded areas representing the standard deviation. The analysis was performed only on the principal angles of each movement. (**a**) SPM analysis (up) and hypothesis test (down) for shoulder flexion/extension during the frontal rise. (**b**) SPM analysis (up) and hypothesis test (down) for shoulder abduction/adduction during the lateral rise. (**c**) SPM analysis (up) and hypothesis test (down) during the reaching task. (**d**) SPM analysis (up) and hypothesis test (down) for elbow flexion/extension during the reaching task.

The results of SPM waveform analysis are displayed in Figure 3 and Figure 4 for healthy weight and obese groups, respectively. In the healthy weight group, statistically significant differences were found near the peaks of shoulder angles in all tasks, whilst no significant difference was found for elbow flexion/extension in the reaching task. In the obese group, a statistically significant difference was found only near the peaks of shoulder abduction/adduction in lateral rise.

## 4. Discussion

The aim of this study was to validate an IMU-based experimental set-up for the quantitative measurement of upper limb kinematics in obese subjects, to compare it with respect to the gold standard implemented using an optoelectronic system and also considering healthy weight subjects as control group; furthermore, the study aimed to identify any possible functional limitations in obese subjects compared to healthy subjects and evaluate whether the IMU-based approach was able to discriminate these differences.

Focusing on ROMs, the comparison between MoCap and IMU-based systems reported a moderate-to-strong correlation for shoulder principal rotation angle in each task and for elbow flexion/extension in reaching task in healthy subjects, whilst only a moderate correlation was found in obese individuals. In both groups, no correlation or only a moderate correlation was reported for non-principal joint rotation angles. 

Concerning the agreement analysis, BA plots for the principal angles of each task reported the presence of a bias for both groups, higher in OG; in addition, BA plots presented wide limits of agreement, especially for the obese group. Similar results on healthy-weight individuals were reported by Henschke et al. [49] who obtained a considerable bias and wide limits of agreement in shoulder abduction/adduction and shoulder flexion/extension when comparing MoCap and IMU measurements. The same issues were also reported by Bravi et al. [50] when comparing IMU and goniometric measurements of shoulder flexion/extension and abduction/adduction in both healthy individuals and patients with cervical spinal cord injury; however, these similarities should be taken with caution due to the differences in both experimental set-up and study population. Regarding the estimation shoulder abduction/adduction, errors may arise from STA. In fact, when the shoulder abducts, the scapula can glide over 10cm beneath the skin, displacing the sensor on the scapular bone and thus introducing measurement errors [39,51]. The risk of unwanted sensor displacement is higher in individuals with obesity, who have more adipose tissue that may affect the scapula movements. It should also be noted that the accuracy and consistency of joint movement estimates using IMUs are influenced by task complexity, which can impact the variability of out-of-plane shoulder joint angles, such as abduction/adduction [39,52]. 

With respect to waveform analysis, SPM highlighted a similar trend between the measurements obtained with the MoCap and the IMU-based system in the healthy weight group. On the other hand, statistically significant differences were found at either the maximum or the minimum values of shoulder angle in each task. Conversely, statistically significant differences were found only at positive and negative shoulder abduction/adduction peaks in lateral rise for the obese group. However, it is worth noting that further differences might be disguised to some extent by the high dispersion of the data, as highlighted by the standard deviation values. Concerning elbow flexion/extension angle during reaching, no differences were found in any group. Similar results have been reported by Goreham et al. [53] for upper limb motion in healthy subjects; in this study, the authors hypothesized that waveform differences could be due to kinematic cross-talk, as well as to gimbal lock, and skin motion artefacts. Regarding cross-talk, according to the literature, IMU-based joint-angle measurement accuracy depends not just on the specific joint under and the motion task, but also on the employed IMU data processing [54]. According to a recent literature review [39], the most precise IMU-based joint angles are obtained by exploiting kinematic constraints on gyroscope data to refine joint axis definitions. Together with a magnetometer-free sensor fusion algorithm, this approach yielded an RMSE between IMU and MoCap below 2° during elbow flexion/extension [40]. In the current study, the XSens MVN model was used to calculate IMU angles. During elbow flexion/extension, the RMSE values were comparable to those reported by Humadi et al. [55], who used the same model. It can be hypothesized that such a model may not be able to effectively offset the technological error. However, it is also important to acknowledge that despite the attempt to reduce skin motion and soft tissue artefacts by using rigid structures and elastic bands for sensor attachment on subjects, their effect cannot be disregarded, particularly in individuals with obesity. During the reaching task, the presence of the cluster on the arm could have influenced the movement and thus the angle estimation.

Shifting our focus on the clinical importance of the presented approach, the comparison of the angular outputs between healthy weight and obese subjects demonstrated a possible functional limitation in obese subjects. In shoulder flexion/extension in frontal rise and reaching, and in shoulder abduction/adduction in lateral rise, the ROMs of the obese group were lower than the healthy weight group. Interestingly, the inertial system did not detect differences in shoulder abduction/adduction angles, confirming its lower ability to discriminate with respect to the MoCap system in terms of clinical relevance; no statistically significant differences were found for elbow flexion/extension for IMU. Concerning arm ROM, similar results have been reported by Cau et al. [23] when comparing the ROMs of upper limbs in obese and healthy weight women during several movements mimicking some basic occupational tasks; the authors found a statistically significant difference for arm elevation ROMs in frontal and lateral rise, where ROMs in obese group presented lower values than what obtained in the healthy weight group. However, it is necessary to underline that a different protocol was used for marker placement and angle computation, so caution should be taken when making comparisons. 

The main limitations of this study can be related both to the chosen experimental and the analytical approach. It should be noted that due to the complexity of shoulder joint, it is not easy to uniquely estimate its rotation angles. To compute shoulder angles, rotation matrices were computed according to the MVM model provided by the manufacturer, presenting its own degrees of freedom and functional constraints. The adoption of such a model may have resulted in an underestimation of the actual shoulder joint rotation angles [38]. In addition, as previously stated, only the four central repetitions out of the 6 performed were considered for the analysis of each task. Although this choice reduced the variability in performing the gesture, it was difficult for the subjects to return to the exact same position at the end of each repetition, resulting in the following repetition starting from a different position. Unfortunately, it was not also possible to assess the repeatability of the measurements, since obese patients became easily tired during the tests and we had to guarantee their overall comfort and safety. 

Apart from BMI, it should also be considered that the comparison between obese and healthy weight individuals was conducted on two non-age-matched groups, and this discrepancy is due to the availability of patients in the hospital during the testing period. Although there is evidence that individuals with high BMI present reduced joint mobility [56], it should also be noted that age can also affect the true joint mobility and increase skin looseness [57,58,59], and thus increase the presence of STA. In addition, fat distribution can also vary between genders and thus affect body morphometry [60], and influence mobility.

Other limitations could be related to the performed analysis that was restricted only to the shoulder and elbow principal angles and to a very limited set of functional tasks. In fact, frontal and lateral rises are simple motions that develop only on the sagittal and frontal plane, respectively, whilst reaching was performed only at a fixed height and distance of the object. The investigation of movements in daily-life activities would represent an important step toward the validation of inertial sensors for upper limb kinematics assessment and its usage in several applications. In addition, the extension of the analysis to the estimation of body segments’ velocity and acceleration would be interesting to have a more complete evaluation of the motion. Furthermore, the development of modelling techniques to reduce the impacts of skin motion and soft tissue artefacts might also improve the applicability on obese subjects. 

Finally, further studies with a higher number of participants, matched for age and sex, would make it possible to increase the knowledge about upper-limb functional limitations in an obese population.

## 5. Conclusions

Upper-limb motion analysis is crucial for identifying potential function limitations and monitoring the progress of rehabilitation treatments. IMUs represent a promising solution with several advantages over traditional MoCap systems, such as the ability to monitor patients in real-life contexts while preserving the ecological validity of their movements. The results of this study partly validated the correlation and concordance between the assessed IMU-based system and the MoCap system, regarding the estimation of shoulder and elbow principal angles in the explored motor tasks. According to the results, the presence of bias and wide limits of agreement may not allow the use of the inertial system in clinical evaluations, where accuracy is critical for identifying clinically relevant thresholds. Furthermore, the IMU-based system demonstrated poorer performance in identifying functional limitations in the obese participants while executing the assigned motor tasks. Despite the presented limitations, it should be noticed that, to the best of our knowledge, this research is one of the first studies assessing the performance of IMU-based system in estimating upper joint angles. Further research is necessary to improve the quality of IMU-based measurements and increase the knowledge of possible alterations of motion patterns in obese individuals. For instance, future studies should enhance functional joint axis calibrations, and assess other configurations for the placement of the sensors to better mitigate and reduce STA, particularly at the scapular level. Further research is also required to develop more standardized modelling approaches and data-driven methods for IMU to joint-angle conversion to decrease cross-talk and technological errors. Once the concerns are addressed, implementing an IMU-based set-up would enable the evaluation of upper limb movements in unconstrained environments and in daily-life contexts and also help to monitor and quantify the effects of rehabilitative treatments in outpatient settings.

## Figures and Tables

**Table 1:** Mean anthropometric and clinical features of participants. Values are expressed as mean (SD).

	Obese Group (OG)	Healthy Weight Group (HG)
Participants (M/F)	23 (5/17)	30 (11/15)
Age (years)	46.39 (18.54)	24.76 (2.4)
Body Mass (kg)	112.48 (18.44)	65.66 (9.89)
Height (cm)	163.83 (8.51)	171.38 (8.97)
BMI (kg m^−2^)	41.63 (4.77)	22.29 (2.27)

**Table 2:** Mean and standard deviation values, accuracy, RMSE and Pearson’s correlation coefficient values for the joint angles estimated with the two systems divided per group (OG or HG) and task. ‘*’ = *p*-value < 0.05.

Group	Task	Joint	Movement	MoCap (°)	IMU (°)	Accuracy (%)	RMSE (°)	r
HG	Frontal rise	Shoulder	Flexion/Extension	158.81 (21.09)	153.84 (22.87)	3.13	14.01	0.82 *
	Lateral rise	Shoulder	Abduction/Adduction	119.36 (21.28)	141.16 (24.67)	18.26	26.39	0.80 *
	Reaching	Shoulder	Flexion/Extension	125.29 (9.90)	116.54 (8.87)	6.99	11.50	0.68 *
		Elbow	Flexion/Extension	60.04 (46.01)	50.06 (32.86)	16.62	30.67	0.77 *
OG	Frontal rise	Shoulder	Flexion/Extension	142.25 (29.73)	138.77 (29.21)	7.66	22.9	0.40 *
	Lateral rise	Shoulder	Abduction/Adduction	99.91 (25.54)	129.19 (34.43)	28.31	42.90	0.43 *
	Reaching	Shoulder	Flexion/Extension	101.34 (22.95)	87.05 (23.43)	14.10	28.71	0.40 *
		Elbow	Flexion/Extension	61.30 (37.27)	45.13 (20.66)	26.38	32.11	0.66 *

**Table 3:** Results of the two-way ANOVA. “Patient” (HG or OG) and “system” (MoCap or IMU) were set as factors. The analysis was performed only on the principal angles of each movement.

Task	Movement	Factors	*p*-Value
Frontal Lift	Shoulder Flexion/Extension	Patient	<0.001
		System	0.275
		Patient: system	0.847
Lateral Lift	Shoulder Abduction/Adduction	Patient	<0.001
		System	<0.001
		Patient: system	0.413
Reaching	Shoulder Flexion/Extension	Patient	<0.001
		System	0.001
		Patient: system	0.432
	Elbow Flexion/Extension	Patient	0.803
		System	0.078
		Patient: system	0.674

**Table 4:** Results of the multiple comparisons with Bonferroni correction.

Task	Movement	Group A	Group B	*p*-Value
Frontal Lift	Shoulder Flexion/Extension	HG-MoCap	OG-MoCap	0.017
		HG-IMU	OG-IMU	0.038
Lateral Lift	Shoulder Abduction/Adduction	HG-MoCap	OG-MoCap	0.004
		HG-IMU	OG-IMU	0.129
Reaching	Shoulder Flexion/Extension	HG-MoCap	OG-MoCap	< 0.001
		HG-IMU	OG-IMU	< 0.001

## Data Availability

Data available on request due to restrictions, e.g., privacy or ethical.
